# Therapist-Guided Verbalization During Tabletop Cognitive Tasks to Support Self-Monitoring and Daily Functioning After Stroke: A Case Report of Two Cases

**DOI:** 10.7759/cureus.111226

**Published:** 2026-06-21

**Authors:** Ryo Kanda, Akira Kudo, Shunichi Sugihara, Fumie Shinmei, Yusuke Fujiwara

**Affiliations:** 1 Department of Rehabilitation, Sapporo Shuyukai Hospital, Sapporo, JPN; 2 Department of Neurosurgery, Sapporo Shuyukai Hospital, Sapporo, JPN

**Keywords:** cognitive rehabilitation, executive dysfunction, self-monitoring, stroke rehabilitation, therapist-guided verbalization

## Abstract

Executive dysfunction after stroke can impair self-monitoring and interfere with daily functioning. Tabletop cognitive tasks are commonly used in cognitive rehabilitation, but transferring training effects to daily life remains a challenge. This case report describes two patients with post-stroke executive dysfunction who received tabletop cognitive interventions incorporating therapist-guided verbalization and self-monitoring. Although the task content and intervention structure differed between the cases, both interventions used task performance as an opportunity to promote verbalization of the thinking process, reflection, error detection, and self-monitoring. Following the intervention period, both patients showed improvements in task performance and daily functioning. In Case 1, the patient became able to complete the Trail Making Test Japanese Version (TMT-J), and goal-directed behavior in daily life improved. In Case 2, TMT-J Part B became achievable, the Kohs Block Design Test Japanese Version improved, medication errors were no longer recorded, and improvements were also observed in other instrumental activities of daily living. These cases suggest that therapist-guided verbalization and self-monitoring during tabletop cognitive tasks may be a clinically feasible approach for supporting the transfer of cognitive rehabilitation strategies to real-life activities, although causal conclusions cannot be drawn from this report of two cases.

## Introduction

Executive dysfunction, including impairments in goal-directed behavior, inhibition, cognitive flexibility, and self-monitoring, is frequently observed after a stroke and may interfere with independent living [1,2]. In particular, instrumental activities of daily living, such as medication management, shopping, and community mobility, require the integration of multiple cognitive processes and are, therefore, vulnerable to the effects of executive dysfunction. Impaired self-monitoring and reduced awareness of deficits may further limit patients’ ability to recognize errors, modify their behavior, and apply strategies to daily activities [2,3].

In cognitive rehabilitation for post-stroke cognitive impairment, tabletop cognitive tasks are often used because they are easy to implement in clinical settings. However, improvement in tabletop task performance does not necessarily generalize to daily life, and transferring cognitive improvements to real-life activities remains a major challenge [1,4,5]. To address this problem, approaches that emphasize metacognitive strategy use, self-monitoring, and transfer, such as the Multicontext Approach, have been proposed [6,7]. Other metacognitive interventions, such as Goal Management Training, also emphasize stopping ongoing behavior, monitoring goals, and applying strategies to everyday tasks [8]. These approaches are clinically relevant because they encourage patients to monitor their own performance, recognize errors, and apply strategies beyond the training context.

However, in inpatient rehabilitation settings, practicing across multiple task contexts or applying abstract strategies may be difficult, particularly for patients with impaired self-monitoring or reduced awareness. One practical approach is to use tabletop cognitive tasks not merely as exercises for task performance, but also as opportunities for therapists to promote verbalization of the thinking process and self-monitoring. By verbalizing task goals and reasons for their judgments and reflecting on their own responses, patients may practice pausing before acting, noticing discrepancies between intended and actual performance, and correcting errors. In this way, therapist-guided verbalization during tabletop tasks may provide a clinically feasible bridge between cognitive task practice and strategy use in daily activities.

The purpose of this study was to describe the clinical use of therapist-guided verbalization and self-monitoring during tabletop cognitive tasks and the associated changes in task performance and daily functioning in two patients with post-stroke executive dysfunction. Both patients provided consent for publication, and institutional requirements for case reports were followed.

## Case presentation

Methodological approach

The following two cases were retrospectively summarized from clinical records during inpatient rehabilitation. Both patients were treated conservatively for ischemic stroke and received inpatient rehabilitation. Both patients were considered suitable for this therapeutic approach because they were able to participate in tabletop cognitive tasks and follow simple task instructions, while showing difficulties in maintaining task goals, monitoring their own performance, detecting errors, and/or applying strategies to daily activities. Within each case, the same type of tabletop cognitive tasks was used across the initial conventional cognitive rehabilitation period and the subsequent verbalization-based or transfer-focused intervention period, while the main change in the intervention was the quality and structure of therapist cueing. Cognitive task performance was assessed using the Trail Making Test, Japanese Version (TMT-J) [9,10] and the Kohs Block Design Test, Japanese Version [11,12]. The TMT-J was used to assess visual attention, processing speed, and cognitive flexibility, whereas the Kohs Block Design Test Japanese Version was used to assess visuospatial construction and nonverbal reasoning. Both tests were administered using officially purchased Japanese versions in accordance with the manuals and institutional procedures. Daily functioning was evaluated based on clinical observations, rehabilitation records, nursing records related to medication management, performance during medication management practice, information from temporary home stays when available, and reports from the patient and family. Improvements in instrumental activities of daily living were, therefore, interpreted from multiple clinical sources rather than from a standardized Lawton Instrumental Activities of Daily Living (IADL) scale.

Intervention sessions were provided as part of routine inpatient rehabilitation approximately five times per week. Each session lasted approximately 40-60 minutes. Therapist cueing was adjusted according to each patient’s task performance and consisted of prompts to verbalize the task goal, the next step, the reason for the patient’s judgment, possible errors, and self-checking strategies. Progression was based on clinical judgment, including the patient’s ability to maintain task rules, verbalize the task goal, detect errors, correct responses with reduced cueing, and apply the self-checking strategy to daily activities. Because the interventions were delivered in routine clinical practice, formal intervention fidelity procedures were not used.

Case 1

Case 1 was a 64-year-old right-handed man with multiple small ischemic lesions in the bilateral frontoparietal subcortical white matter, mainly involving the centrum semiovale and corona radiata (Figure 1A). Although mild left hemiparesis was observed, it did not cause marked impairment in basic activities of daily living, and the patient was able to walk independently.

**Figure 1:**
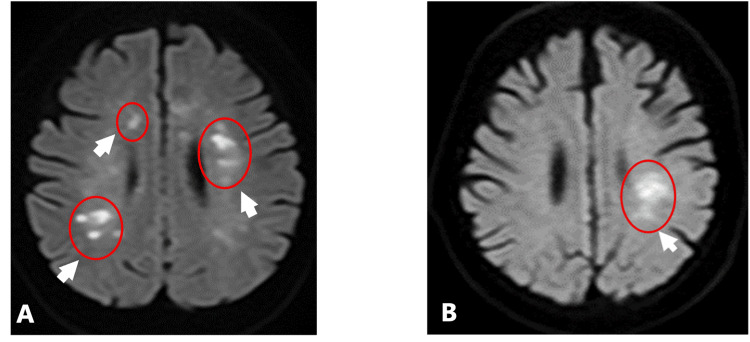
Diffusion-weighted magnetic resonance images of the two cases (A) Case 1: Representative axial diffusion-weighted images showing multiple small ischemic lesions in the bilateral frontoparietal subcortical white matter, mainly involving the centrum semiovale and corona radiata. (B) Case 2: Representative axial diffusion-weighted images showing an ischemic lesion in the left deep white matter, mainly involving the corona radiata.

After stroke onset, the patient was able to understand single words and simple direct questions, suggesting that basic comprehension was relatively preserved. However, he had difficulty retaining and manipulating instructions that contained multiple elements, maintaining task rules, and monitoring his own responses during task performance. He was also unable to complete the TMT-J or the Kohs Block Design Test Japanese Version. This difficulty was considered more likely to reflect impairment in frontal executive processes, including working memory and manipulation of verbal information, than a primary aphasic comprehension deficit.

In daily life, behaviors suggestive of impaired goal-directed behavior were observed, such as walking into the hallway barefoot and returning from the toilet without completing the intended action. In addition, when behavioral errors were pointed out, he frequently gave defensive or confabulatory explanations, suggesting impaired self-monitoring.

Rehabilitation was initiated soon after stroke onset, and conventional cognitive rehabilitation was provided during the first month. From the second month after stroke onset, a verbalization-based tabletop intervention was introduced approximately five times per week. The intervention used tabletop cognitive tasks that required the manipulation of multiple elements, including logic puzzles, mental rotation tasks, and Sudoku. During task performance, the therapist encouraged the patient to verbalize his thought processes, reflect on his responses, and detect and correct errors. For example, prompts such as “What will you do next?", “Why do you think so?" and “Can you check whether this is correct?” were used to help the patient clarify the task goal, explain his reasoning, and notice and correct errors. Cueing was provided when the patient had difficulty maintaining the task goal, explaining his reasoning, or detecting errors. As the patient became more able to verbalize the task goal, explain the reason for his responses, and check his own performance, the therapist encouraged him to perform these self-monitoring steps with less direct prompting.

After the intervention, the patient became able to complete the TMT-J and the Kohs Block Design Test Japanese Version, and daily functioning also improved. Behaviors suggestive of impaired goal-directed behavior were no longer observed, and independent living at home became a realistic goal (Table 1). At outpatient follow-up after discharge, the patient was living without major difficulty, and the improvement in daily functioning was maintained.

**Table 1:** Summary of neuropsychological and functional outcome changes across the two cases For Case 1, “before verbalization-based intervention” refers to the assessment at approximately one month after stroke onset, immediately before the introduction of the verbalization-based tabletop intervention, and “after intervention” refers to the assessment approximately two months after stroke onset. For Case 2, “before transfer-focused intervention” refers to the assessment at the end of Phase A, immediately before the introduction of the three-step transfer-focused intervention, and “after intervention” refers to the assessment at the end of Phase B. TMT-J: Trail Making Test Japanese Version; IADL: instrumental activities of daily living.

Outcome domain	Case 1: Before verbalization-based intervention	Case 1: After intervention	Case 2: Before transfer-focused intervention	Case 2: After intervention
TMT-J Part A	Not completed	81 s	218 s	194 s
TMT-J Part B	Not completed	111 s	Not completed	340 s
Kohs Block Design Test Japanese Version	Unable to perform	IQ-equivalent score 110	IQ-equivalent score 58	IQ-equivalent score 82
Main daily-life problem	Impaired goal-directed behavior	Improved goal-directed behavior	Medication errors and IADL difficulty	Medication errors resolved; IADL improved
Functional status	Institutional discharge considered	Independent living at home became realistic	Supervision or assistance required for IADL	Independent or minimal assistance for IADL

Case 2

Case 2 was a 79-year-old man who presented with post-stroke cognitive impairment, including mild aphasia, reduced attention, and executive dysfunction, after an ischemic stroke. No marked motor impairment was observed, and he was physically independent in basic activities of daily living. Diffusion-weighted magnetic resonance imaging showed a hyperintense ischemic lesion in the left deep white matter, mainly involving the corona radiata. The prefrontal cortex itself was relatively preserved (Figure 1B).

**Figure 2:**
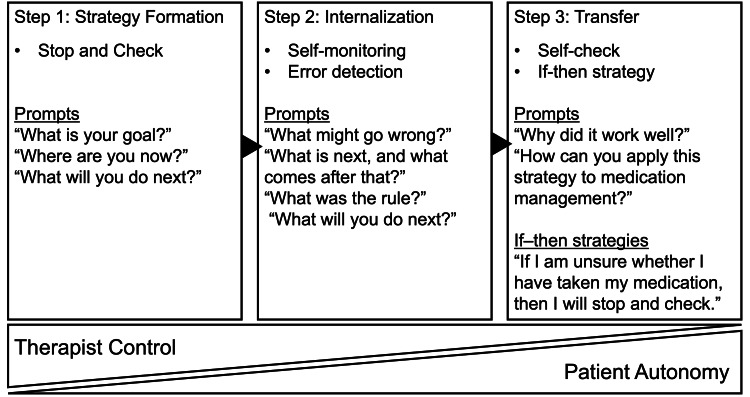
Three-step structure of the transfer-focused intervention in Case 2 The intervention progressed from therapist-led cueing to establish a self-checking strategy (Step 1), to reduced cueing and patient-led self-monitoring (Step 2), and finally to explicit transfer of the strategy to medication management and other daily activities (Step 3).

During hospitalization, the patient showed marked difficulty with medication management, and missed doses and duplicate dosing were observed. During a temporary home stay, difficulties were also reported in other instrumental activities of daily living, including shopping, using public transportation, and preparing for bathing.

The intervention followed an A-B design and was initiated soon after stroke onset during inpatient rehabilitation. Phase A consisted of approximately four weeks of conventional tabletop cognitive training beginning soon after stroke onset, and Phase B consisted of approximately four weeks of a structured transfer-focused intervention thereafter. Intervention sessions were conducted approximately five times per week. The same tabletop tasks were used throughout both phases, and only task difficulty was adjusted according to the patient’s performance. The tasks included a number search task and a card sorting task, which targeted attentional shifting, rule maintenance, inhibition, and self-monitoring.

During Phase A, conventional training was provided without structured strategy cueing. In contrast, during Phase B, a transfer-focused intervention composed of three sequential steps was introduced to promote the formation, internalization, and transfer of a self-checking strategy to daily activities (Figure 2). In Step 1, therapist-led cueing was used to help the patient stop and verify the task goal and current performance. In Step 2, therapist cueing was gradually reduced to encourage patient-led self-monitoring and error detection. In Step 3, the self-checking strategy was explicitly linked to medication management and other daily activities using if-then statements. Progression across the three steps was based on clinical judgment rather than predetermined numerical criteria. Therapist cueing was reduced when the patient became able to restate the task goal, identify the next action, detect errors, and correct responses with less assistance. Transfer to medication management and other daily activities was introduced when the patient could use the self-checking strategy during tabletop tasks with reduced therapist support.

After the introduction of the transfer-focused intervention, improvements were observed in both task performance and daily functioning. The patient became able to complete TMT-J Part B, and the Kohs Block Design Test Japanese Version also improved. In daily life, medication errors were no longer recorded in nursing and rehabilitation records during medication management practice, and improvements were also observed in shopping, using public transportation, and preparing for bathing based on clinical records from temporary home stays and reports from the patient and family (Table 1). Interviews with the patient and his family one month after discharge indicated that these improvements were maintained.

Intervention characteristics common to the two cases

Although the interventions were not identical, the common feature across both cases was that tabletop cognitive tasks were used to promote verbalization of the thinking process and self-monitoring. In both cases, tabletop tasks were used not merely as cognitive exercises, but also as opportunities for the patients to explain their reasoning, reflect on their responses or actions, and detect and correct errors.

On the other hand, the two cases differed in the degree of structure and the explicitness of transfer to daily life. In Case 1, an exploratory intervention was provided using reasoning-based tasks, including logic puzzles, mental rotation tasks, and Sudoku, to promote self-monitoring through verbalization and reflection. In Case 2, the intervention was developed into a more structured transfer-focused approach, in which the same tabletop tasks were continued while therapist-provided verbal cues were gradually adjusted to support the formation, internalization, and transfer of a self-checking strategy to medication management.

Thus, the common therapeutic element across the two cases was not the specific tabletop task itself, but the use of task performance as a medium to promote verbalized reasoning and self-monitoring. Changes in neuropsychological and functional outcomes before and after the verbalization-based intervention or transfer-focused intervention are shown in Table 1.

## Discussion

This report described two patients with post-stroke executive dysfunction who underwent tabletop cognitive interventions incorporating therapist-guided verbalization and self-monitoring, with subsequent improvements in task performance and daily functioning. Because this report included only two patients and both were in the early post-stroke recovery period, the observed improvements should be interpreted as associations rather than evidence of a causal intervention effect. Below, we discuss possible mechanisms underlying these changes and their relevance to transfer to daily life.

Self-monitoring and awareness of deficits are important factors related to rehabilitation outcomes after acquired brain injury [2]. In the present cases, asking the patients to verbalize the task goal, the next step, the reason for their judgment, and possible errors created opportunities for them to pause before acting and compare their ongoing actions with the intended goal. This process may have helped patients notice discrepancies between intended and actual performance and may have supported error detection and self-correction. Therefore, verbalization may have functioned not merely as a communication tool but as a therapeutic tool to support working memory, self-monitoring, and goal-directed behavior. This interpretation is consistent with metacognitive interventions such as goal management training, which emphasize stopping ongoing behavior, monitoring goals, and applying strategies to everyday tasks [8]. It is also consistent with models of awareness deficits that emphasize the importance of self-monitoring and feedback during task performance [3]. However, because self-monitoring, error detection, self-correction, and cue dependency were not objectively measured over time, this mechanism should be interpreted as a clinically plausible explanation rather than a demonstrated mediation process.

The clinical course of Case 1 may support this interpretation. Before the intervention, the patient had difficulty understanding instructions that contained multiple elements and maintaining task rules, but after the intervention, he became able to complete the TMT-J and the Kohs Block Design Test, Japanese Version. In addition, behaviors suggestive of impaired goal-directed behavior were no longer observed in daily life. Although the influence of spontaneous recovery cannot be excluded, the pattern of improvement was consistent with the therapeutic targets of the intervention, namely executive function, self-monitoring, and goal-directed behavior.

Case 2 provided implications regarding transfer to daily activities. The same tabletop tasks were used in both phases, whereas therapist cueing, cue fading, patient-led verbalization, self-monitoring, and application of the strategy to medication management and other daily activities were introduced stepwise during Phase B. Improvements in the Kohs Block Design Test Japanese Version, TMT-J Part B, medication management, and other instrumental activities of daily living during and after Phase B suggested that explicitly linking self-monitoring strategies practiced during tabletop tasks to daily activities may support transfer to real-life performance.

Although the present approach was not a formal implementation of goal management training or the multicontext approach, it shared several core principles with these established metacognitive rehabilitation approaches. Specifically, the intervention emphasized pausing before action, verbalizing task goals and next steps, monitoring ongoing performance, detecting errors, and linking strategies practiced during cognitive tasks to daily activities. In this sense, therapist-guided verbalization during tabletop cognitive tasks may be understood as a clinically feasible way to incorporate metacognitive strategy training into routine inpatient rehabilitation.

These findings are related to the challenge of transfer in cognitive rehabilitation. Cognitive training may improve performance on trained or related tasks, but generalization to daily activities is often limited [1,4,5]. In the present cases, tabletop tasks were used as a medium for practicing monitoring, reflection, error detection, and strategy use. Therefore, the therapeutic value of tabletop cognitive tasks may lie not only in the tasks themselves but also in how therapists use task performance to structure patients’ thinking and support self-monitoring and strategy transfer to daily activities.

From a clinical perspective, therapist-guided verbalization may help therapists connect cognitive task performance with strategy use in daily activities. For patients to monitor their own performance and use strategies in daily activities, prompts tailored to their performance and reflection before and after actions are necessary. Because this method does not require specialized equipment and can be incorporated into commonly used tabletop cognitive tasks, it may be feasible in inpatient rehabilitation settings.

Several limitations should be considered. First, this report included only two cases, and causal relationships between the intervention and the observed improvements cannot be established. Second, both patients were in the early post-stroke recovery period, and spontaneous recovery may have substantially contributed to the observed improvements. In addition, both patients continued to receive conventional inpatient rehabilitation, and repeated exposure to similar tabletop tasks, environmental support, and increased familiarity with hospital routines may also have influenced the outcomes. Therefore, the observed changes may not be attributable solely to verbalization-based or transfer-focused interventions. Third, the interventions were not identical between the two cases, and the findings should not be interpreted as evidence for a single fixed intervention protocol. In addition, formal intervention fidelity procedures, such as independent monitoring of cueing consistency or adherence to a predefined protocol, were not used because the interventions were delivered as part of routine clinical practice. Fourth, the proposed mechanism of improved self-monitoring through therapist-guided verbalization was inferred from clinical observations and changes in task performance and daily functioning, but objective process-based measures were not available. For example, the frequency of therapist cues, spontaneous verbalization, self-checking, error detection, self-correction, and cue dependency was not systematically recorded over time. Therefore, this report cannot determine whether changes in these processes mediated the observed improvements in task performance or daily functioning. Fifth, functional outcomes were evaluated using clinical records, observations, and patient and family reports rather than standardized IADL scales, which may limit the objectivity and reproducibility of the functional outcome assessment. Finally, in Case 2, medication management was limited to daily management for safety reasons, and family support was planned after discharge; therefore, uncertainty remains regarding generalization to fully independent multi-day medication management.

Future studies should examine the reproducibility of these findings using larger samples, standardized intervention procedures, and prospectively recorded process-based measures such as verbalization, self-checking, error detection, cue dependency, and self-correction. Such studies may clarify whether changes in these processes are associated with improvements in daily functioning and how tabletop cognitive tasks can be used not merely as cognitive training tasks but as structured opportunities to promote self-monitoring and transfer to daily life.

## Conclusions

In this report of two cases, tabletop cognitive tasks incorporating therapist-guided verbalization and self-monitoring were associated with improvements in task performance and daily functioning after stroke. Because both patients were in the early post-stroke recovery period and no objective process-based measures of self-monitoring were systematically recorded, causal conclusions cannot be drawn. These cases suggest that using tabletop cognitive tasks as opportunities to practice therapist-guided verbalization and self-monitoring may be a clinically feasible approach for supporting the transfer of cognitive rehabilitation strategies to daily activities in patients with post-stroke executive dysfunction.
